# Three-dimensional organotypic mouse brain slices to study Alzheimer’s disease pathologies: a review

**DOI:** 10.3389/frdem.2025.1585124

**Published:** 2025-07-25

**Authors:** Christian Humpel

**Affiliations:** Laboratory of Psychiatry and Experimental Alzheimer’s Research, Medical University of Innsbruck, Innsbruck, Austria

**Keywords:** Alzheimer’s disease, organotypic 3D culture, brain slice, pathology, review

## Abstract

Alzheimer’s disease (AD) is a severe neurodegenerative brain disorder molecularly characterized by extracellular β-amyloid plaques, intraneuronal tau neurofibrillary tangles, cholinergic neuron death, neuroinflammation, vascular damage, and astroglial and microglial activation. AD is a complex disorder, with >99% of all cases being sporadic and typically occuring around the age of 65. Due to this intricate nature of the disorder, *in vitro* experiments have limitations; however, three-dimensional organotypic brain slices may offer the best alternative for studying the mechanisms involved in the progression of AD. This review provides an overview of how to study the general aspects of AD *ex vivo*, focusing on (a) β-amyloid plaques in brain slices, (b) tau pathology induced by chemical drugs, (c) cell death of cholinergic neurons and protection by nerve growth factor, (d) activation of astrocytes and microglia, and (e) vascular pathologies, including the role of platelets. Furthermore, we investigated (f) how microcontact printing on brain slices can be used to study the spread of β-amyloid and tau, and (g) how brain slices can help identify novel human AD biomarkers.

## Organotypic brain slices and Alzheimer’s disease

Alzheimer’s disease (AD) is a severe neurodegenerative brain disorder, characterized by two main pathologies, namely extracellular β-amyloid plaques and intraneuronal neurofibrillary tangles (NFTs) composed of phosphorylated Tau (Braak and del Tredici, 2015; Swerdlow, 2012). However, AD is a complex disease with many other pathologies such as neuroinflammation, silent strokes, activation of astrocytes and microglia, and dysfunction of microglia (Irvine et al., 2008; Obulesu and Jhansilakshmi, 2014; Glass and Arnold, 2012). There is also severe vascular damage, with the breakdown of the blood–brain barrier (BBB), vascular damage, angiogenesis, and activation of platelets, as well as deposition of β-amyloid in vessels, known as cerebral amyloid angiopathy (CAA) (Perlmutter, 1994; Greenberg et al., 2020). Finally, cholinergic neurons degenerate in the brain, and the loss of acetylcholine results in severe memory defects (Berry and Harrison, 2023; Dunnett and Fibiger, 1993).

Therefore, studying this complex disorder is challenging. AD is a disease of old age (>65 years) and is a non-genetic sporadic disease (Braak and del Tredici, 2015; Glass and Arnold, 2012). The exact cause for the emergence of AD is not yet fully understood. However, many risk factors are known, such as lifestyle factors, that affect vascular brain support. Sporadic AD is the most common form of AD (>99%), and only a few families have a genetic form of AD (1% of cases) (Braak and del Tredici, 2015; Glass and Arnold, 2012). As mentioned, the analysis of human brains would be the best strategy to study AD using brain imaging [positron emission tomography (PET) or magnetic resonance tomography (MRT)]; however, these techniques do not provide high resolution. Post-mortem brain studies are an alternative, but the complexity of the disease makes it difficult to study, and post-mortem delay results in false-positive results. The human Nun Study (Lemonick and Park, 2001) aimed to overcome this problem, as this is a well-characterized homogenous population. The Nun Study of aging and AD is a continuing longitudinal study, begun in 1986, with the aim to examine the onset and progression of AD in a well-controlled human population (same lifestyle, education, nutrition) with 678 nuns aged 75–106 years. The main outcome was that about one-third of the nuns showed β-amyloid plaques in the brain without any symptoms and with a normal cognitive function, which challenged the β-amyloid-cascade hypothesis (see below) and suggested that lifelong learning might partly counteract the neurodegenerative process.

**Figure 1:**
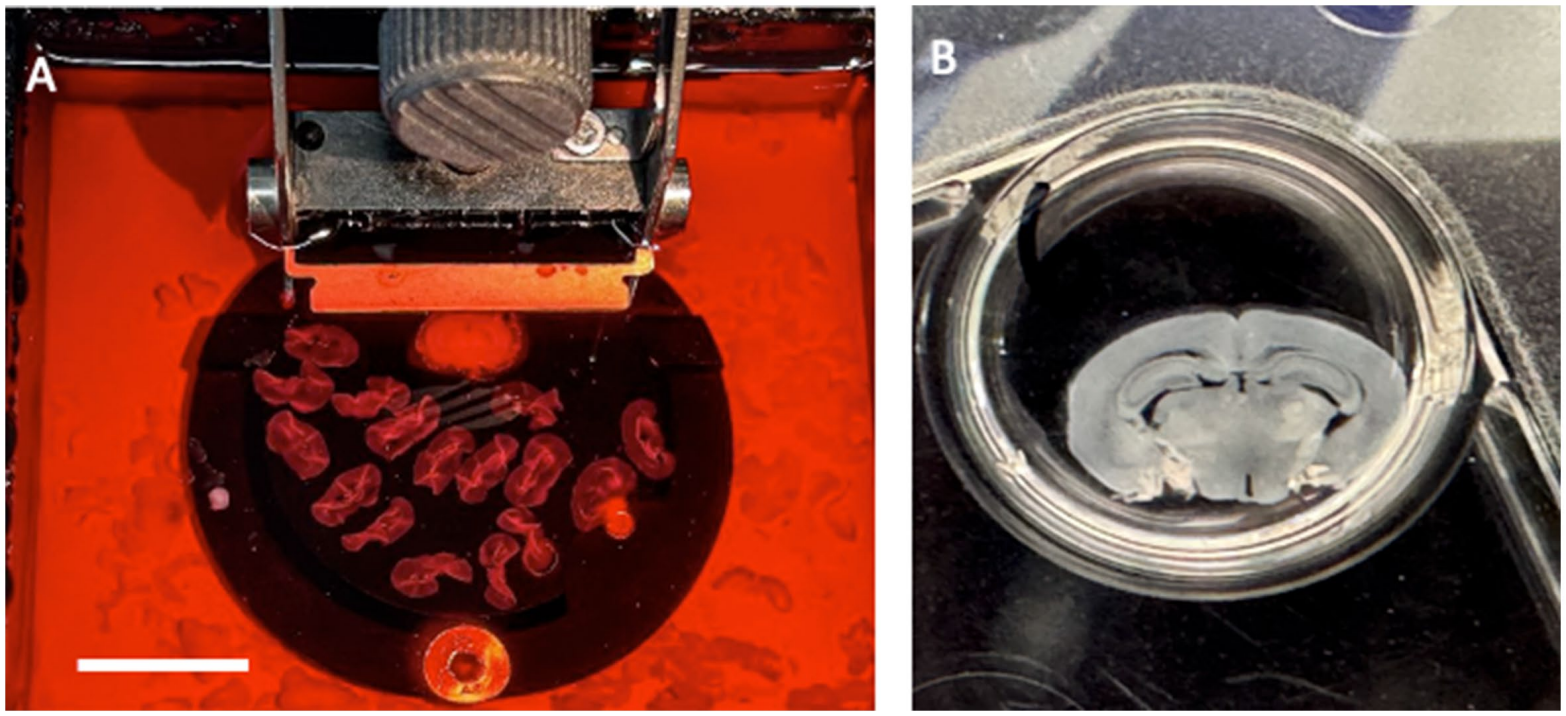
Organotypic brain slices are prepared from a postnatal day 8–10 mouse brain, sectioned with a vibratome 150 μm; **(A)** and then placed on a semipermeable membrane insert and cultured in 6-wells with medium **(B)** for several weeks at 37°C and 5% CO_2_. **(B)** shows a coronal brain slice at the hippocampal level. Scale bar = 2.2 cm **(A)**; 0.5 cm **(B)**.

Animal studies are complex and have primarily focused on transgenic mice (Humpel, 2019). However, transgenic mice do not reflect complex disorders and can only provide information on the progression of plaques or NFTs, but not on the development of sporadic disease. Thus, transgenic mice are useful for studying genetic but not sporadic AD (Foidl and Humpel, 2019a). It would be highly interesting to establish a mouse model of sporadic AD by treating mice for months with risk factors such as drugs that increase blood pressure, glucose, or cholesterol (Korde and Humpel, 2024; Foidl and Humpel, 2019b). Using mouse models is limited, as they live for only 24–28 months. To date, no mouse model of sporadic AD has been established, and we can only study single pathologies using cell culture models. To the best of my knowledge, the organotypic brain slice model is the most suitable for studying different aspects of dementia as it contains all brain cells in a complex three-dimensional architecture (Humpel, 2015). The use of organotypic brain slices began in the 1980s by Gähwiler (1981), improved using membrane inserts by Stoppini’s research (Stoppini et al., 1991), and further developed in my lab using whole coronal vibratome slices (Humpel, 2018) (Figure 1). This review summarizes and discusses how brain slices may be useful for studying different aspects of AD.

### β-amyloid plaques in brain slices

**Figure 2:**
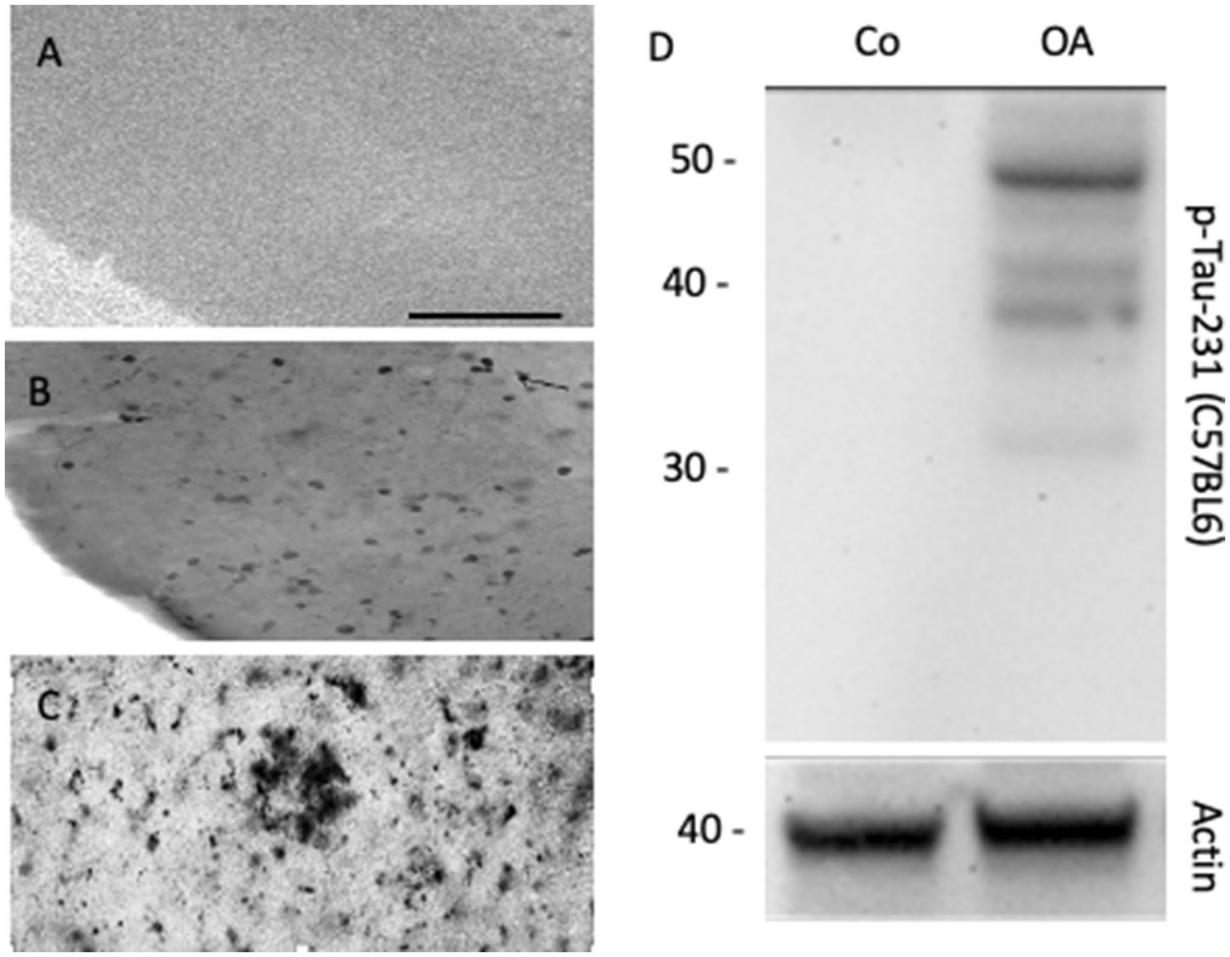
Organotypic brain slices can be cultured from adult APP_SweDI transgenic mice where β-amyloid plaques progressively develop, when incubated from 4-month-old brains **(A)**, 5-month-old brains **(B)**, or 6-month-old brains **(C)**, where β-amyloid plaques are visualized. In organotypic mouse brain slices, the phosphorylation of Tau (e.g., phospho-Tau-231) can be studied when stimulated with okadaic acid (OA) versus a control (Co). **(D)** shows a representative Western Blot, where Tau is seen as a 50 kDa phosphorylated protein after treatment of slices with OA. Actin (40 kDa) serves as a control. Scale bar = 220 μm **(A–C)**.

Aβ is a small peptide of 40 or 42 amino acids. The larger 42 amino acid form is more toxic and tends to form aggressive aggregates, leading to the development of extracellular β-amyloid plaques in the brain, as described in the so-called “β-amyloid cascade hypothesis” (Hardy and Higgins, 1992; Ittner and Götz, 2011; Swerdlow, 2012). The smaller form is deposited in vessels and is found at higher levels in the blood. β-amyloid is formed from a large transmembrane protein, the amyloid precursor protein (APP), which is processed by secretases (LaFerla et al., 2007). Many studies have used transgenic mice with mutated APP that develop β-amyloid plaques (Price et al., 1998; Lannfelt et al., 1993). In our experiments, we used the APP_SweDI mouse model, in which β-amyloid plaques develop in 6-month-old mice (Humpel, 2019), and we aimed to prepare adult brain slices to study the progression of β-amyloid plaques (Humpel, 2019). Unfortunately, a main disadvantage of adult brain slices is that brain cells do not survive well (Humpel, 2019). Usually, slices from postnatal mice flatten and become transparent, which is a good sign for healthy slices. Adult brain slices (and slices taken from mice older than 14 days) do not flatten, and therefore, we cultured thinner (110 μm) slices. Thus, as plaques develop in older mice and not in postnatal mice, it was not possible to study the progression of the plaques, but we could study plaques at different ages of the mice (Figure 2). In another experiment, we showed that neprilysin can degrade these plaques (Humpel, 2022), and slices are useful to study plaque elimination *ex vivo*.

### Tau pathology in brain slices

Tau is a large 60 kDa microtubule-associated protein that plays a role in axonal transport (Gallardo and Holtzman, 2019; Busche and Hyman, 2020). Tau is highly regulated by phosphorylation at about 40 sites. In AD, tau becomes hyperphosphorylated, and this process leads to a defect in axonal transport and thus the neurons die (“Tau-hypothesis of AD”) (Ittner and Götz, 2011; Swerdlow, 2012; Braak and del Tredici, 2012). The reasons for this hyperphosphorylation are unknown; however, enhanced protein kinase activity or decreased phosphatase activity may cause this effect (Bakota and Brandt, 2016; Busche and Hyman, 2020). Furthermore, it is not clear how β-amyloid and tau interact. The slice model may allow the study of transgenic mice with defects in tau protein, leading to NFTs. More importantly, we explored the mechanisms by which tau is phosphorylated and enters the toxicity pathway. Pharmacological treatments should be tested to block hyperphosphorylation, especially of different protein phosphatases. In a previous report, we showed that okadaic acid enhances tau phosphorylation in brain slices (Foidl and Humpel, 2018) (Figure 2D). Okadaic acid is a cytotoxin and was originally isolated from the black sponge *Halichondria okadai*. Amongst others, it particularly leads to the activation of GSK-3β, neuroinflammation, and oxidative stress.

### Cholinergic neurons in brain slices

**Figure 3:**
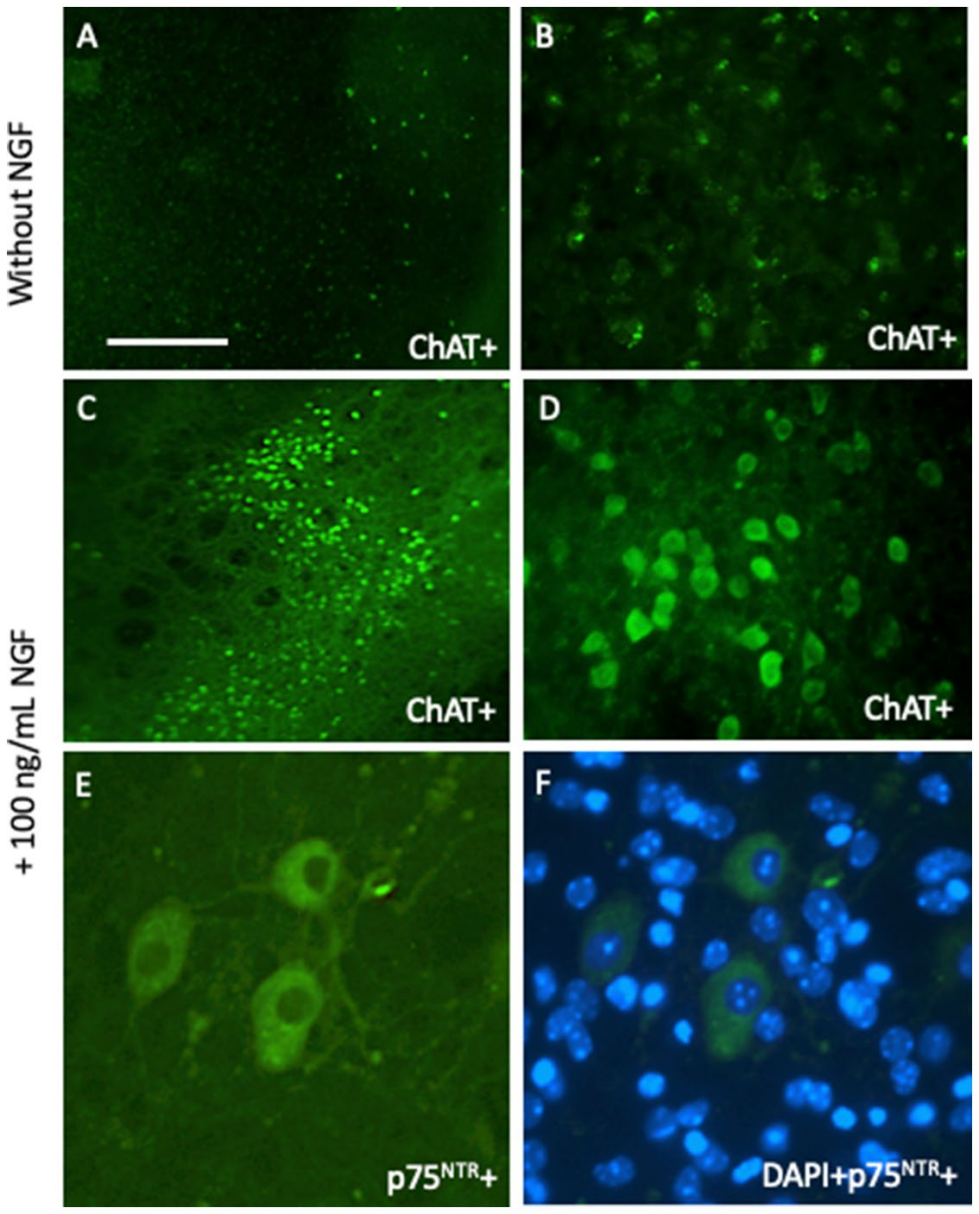
Cholinergic neurons from the basal nucleus of Meynert can be cultured for 2 weeks and degenerate in brain slices without any growth support **(A,B)**, but survive when incubated with 100 ng/mL nerve growth factor (NGF) **(C–F)**. Brain slices are immunohistochemically stained with antibodies against choline acetyltransferase **(A–D)** or the low-affinity NGF receptor p75 neurotrophin receptor (p75^NTR^) **(E,F)**. Scale bar = 660 μm **(A,C)**, 165 μm **(B,D)**, 40 μm **(E,F)**.

Acetylcholine is a major neurotransmitter in the brain that is responsible for memory (Berry and Harrison, 2023; Dunnett and Fibiger, 1993). Cholinergic neurons are located in the basal nucleus of Meynert, where they project to the cortex and are responsible for long-term memory. These neurons are also located in the septum and diagonal band of Broca, project to the hippocampus, and are responsible for short-term memory. Interestingly, cholinergic neurons degenerate in AD, although the reason is unknown (“*cholinergic hypothesis of AD*”) (Berry and Harrison, 2023; Dunnett and Fibiger, 1993). It is well known that the classical growth factor nerve growth factor (NGF) supports the survival of cholinergic neurons (Humpel, 2021). We have a long experience in culturing cholinergic neurons of the basal nucleus of Meynert (nBM) and have shown that NGF supports the survival of these neurons (Lapchak, 1993; Hefti and Will, 1987) (Figure 3). Interestingly, these neurons require long-term support for survival. Thus, the brain slice model may be suitable for studying how growth factors support survival and how pharmaceutical drugs can be developed. Generation of BBB-permeable NGF-like drugs may delay the degeneration of these neurons in the brain.

Cholinergic neurons offer a nice way to study cellular survival. The first issue is to judge if slices flatten and become transparent, as this is a good sign of healthy slices. Thick slices should be deleted. Second, a general blue fluorescent DAPI staining is suggested to evaluate the slice quality and the homogenous distribution of the cellular layer and the orientation of the slices. Third, a fast and simple propidium iodide staining is recommended to see cellular death, as visualized by red fluorescent nuclei. And finally, I suggest specific neuronal markers, like neurofilament or microtubule-associated protein-2, or in the case of cholinergic neurons, choline acetyltransferase. Such cell death assays can be performed with any kind of slices, not only cholinergic neurons. Cholinergic neurons offer a simple way to study cell death as neurons degenerate without NGF and survive with NGF (Humpel and Weis, 2002). In our hands, cholinergic neurons survive for 2–4 weeks with NGF, but we have also seen cholinergic neurons 50 weeks in culture with NGF (Marksteiner and Humpel, 2008).

### Astrocytes and microglia in brain slices

**Figure 4:**
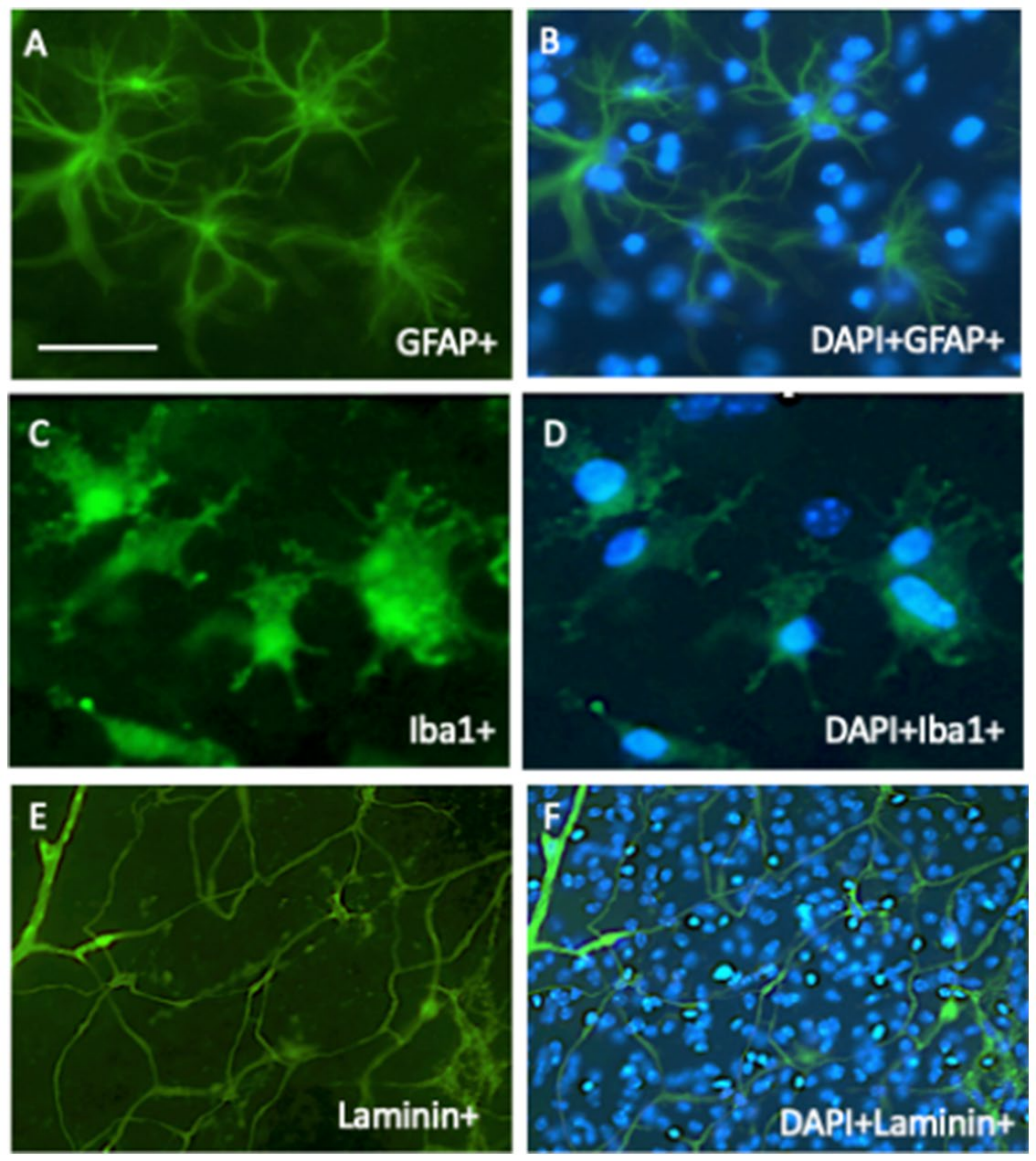
In organotypic brain slices, all brain cells survive and can be studied. In 2-week-old cultured slices, astroglia **(A,B)** survive and are stained with glial-fibrillary acidic protein (GFA), microglia **(C,D)** are stained with Iba1 antibody, and vessel/endothelial cells **(E,F)** with laminin. All cells were stained immunohistochemically with green fluorescent Alexa-488 secondary antibodies. **(B,D,F)** show the counterstaining with blue fluorescent nuclear DAPI. Scale bar = 60 μm **(A–D)**; 260 μm **(E,F)**.

Glial cells are important in the brain and play a role in stabilizing neurons, BBB function, and electrolytic balance (astroglia), formation of myelin sheets along axons, fast signaling (oligodendrocytes), and immune and phagocytic reactions (microglia) (Preeti et al., 2022; Hashioka et al., 2021; Kim et al., 2020; Kim et al., 2018; Schubert et al., 2001; Mrak and Griffin, 1996). These cells are present in cultured brain slices and can be stained for glial fibrillary acidic protein (GFAP, astroglia) (Daschil and Humpel, 2016), myelin oligodendrocyte protein (MOG, oligodendrocytes) (Yilmaz et al., 2024), Iba1, or CD11b (microglia) (Steiner et al., 2024) (Figure 4). In AD, astroglia is found around β-amyloid plaques in the halo and is activated to counteract plaque development and support plaque elimination (Daschil and Humpel, 2016). The main immune cells of the brain are microglia, which are present in different forms: round-amoeboid, ramified, and large macrophages, and their major aim is to eliminate brain debris and plaques (Steiner et al., 2024). Thus, it is of great interest to study microglia, as they become dysfunctional during AD progression. All of these cells can be studied in mouse organotypic brain slices and their respective pathological processes in AD.

### Vascular pathologies and platelets in brain slices

Evidently, vascular structures are damaged in the AD brain, and the smaller β-amyloid (40) peptide is deposited in the brain vessels, causing CAA (Koemans and van Etten, 2025; van den Brink et al., 2024; Godrich et al., 2024; Vinters, 1987). This defect in blood vessels, together with the breakdown of the BBB, causes a loss of energy supply (glucose and O_2_) to the brain and an influx of toxic molecules from the blood. Defects in the vascular structure play a role in the progression of sporadic AD (Scheffer et al., 2021; de la Torre, 2018, 2010; Milionis et al., 2008; Lucas and Rifkind, 2013). It is suggested to maintain healthy vascular brain vessels in order to delay the progression of AD and 14 (vascular) risk factors should be considered: low education, hearing loss, high blood pressure, smoking, fatty food, depression, no sports, diabetes, alcohol, head trauma, air pollution, social isolation, high low-density lipoprotein cholesterol, and visual loss.

Organotypic brain vessels contain the complete vessel network, which can be easily stained with laminin (Figures 4E,F). We studied the role of the vessel system in our laboratory and found an intact vessel that was rearranged and connected in co-cultures. However, we could not prove that the vessels were intact, as they did not transport blood and were not tube-like; thus, we refer to them as vessel-like (Moser et al., 2003). In this context, it is worth mentioning that the major disadvantage of culturing brain slices is the lack of a BBB, and thus, influx and efflux cannot be studied (Humpel, 2015). Vascular structure degenerates in AD, and angiogenic factors can be easily studied, showing re-formation of vessels (Ucar et al., 2020). In addition, vessel repair, such as clotting, can be studied; in this context, the function of platelets is of great interest. Platelets contain much APP and secrete smaller β-amyloid (40) forms that may play a role in blood clotting (Humpel, 2017). It has also been hypothesized that platelets contribute to the pathology of AD. We and others have formed the “vascular hypothesis of AD,” that β-amyloid depositions (formed during CAA) in and around the vessel may spread over into the whole brain (Scheffer et al., 2021; de la Torre, 2018, 2010; Milionis et al., 2008; Lucas and Rifkind, 2013; Humpel, 2011). It has been suggested that damage to brain vessels causes the activation and migration of platelets to repair these vessels, and a dysfunctional cascade may activate the toxic β-amyloid cascade (Humpel, 2017). Such a process can be studied in brain slices: in a previous study, we (Kniewallner et al., 2018) isolated platelets from wildtype and transgenic AD mice, labelled them with a red fluorescent dye, and infused the labelled platelets back into anesthetized wildtype mice. From these mice, brain slices were prepared and cultured. Our data showed that platelets from AD mice severely damaged cortical vessels and entered the parenchyma, causing increased β-amyloid staining, inflammation, and activation of microglia around the lesions.

### Spreading of “prion-like” proteins in slices

It is not clear how the AD pathology starts, but the “spreading hypothesis” (Walsh and Selkoe, 2016) suggests that toxic mutated prion-like proteins (such as alpha-synuclein or β-amyloid or mutated tau) are consumed, taken up by the gut, and transported along the vagus nerve into the brainstem (Wang et al., 2019). As AD develops over the decades, these aberrant/misfolded proteins spread throughout the brain in a prion-like manner (Walker, 2018). It is evident that plaques are already present in young individuals, develop over decades, spread from the brain stem to the entorhinal cortex and hippocampus, and then to the cortex. But how can we study spreading in slices? We have developed a method where proteins or peptides can be delivered onto brain slices loaded into collagen scaffolds (Ucar and Humpel, 2018). When we studied the survival of cholinergic neurons, growth factors (in this case, NGF) were added to the medium. This was never a problem, as long as the growth factors diffuse through the semipermeable 0.4 μm membranes and are absorbed into the brain slices. However, in some experiments, it was necessary to load the factors of interest directly onto the brain slices. The pipetting of a 1 μL solution directly onto the slice is not successful, as the fluid rapidly diffuses all over the brain slice. Thus, to apply the substance locally, we developed and optimized the collagen scaffold. Collagen is a natural, biocompatible, nontoxic molecule that can aggregate when mixed with 4-arm-poly (ethylene glycol) (PEG) succinimidyl succinate (PEG) (Ucar and Humpel, 2018). Very small collagen scaffolds with a diameter of 1 mm can be produced and loaded with any molecule of interest. These scaffolds can then be placed directly onto the brain slice in any area of interest (Ucar and Humpel, 2018). The collagen scaffold degrades over time and slowly releases the substance of interest. Using these collagen scaffolds, we applied β-amyloid or tau, or alpha-synuclein onto brain slices and studied their spreading in the brain slice (Korde and Humpel, 2022; Ucar et al., 2022; Moelgg et al., 2021).

### Identify novel human AD biomarkers using microcontact printing

**Figure 5:**
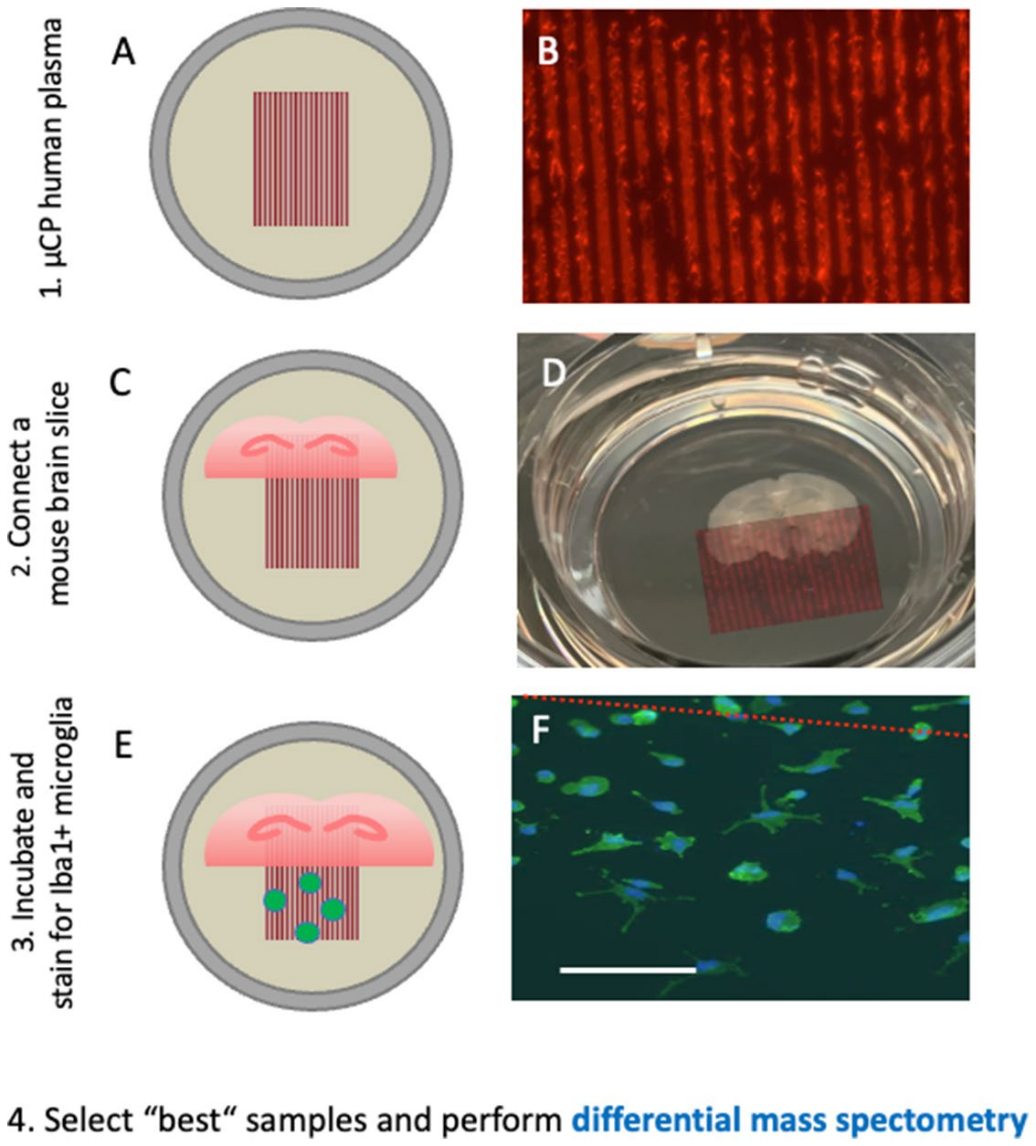
Microcontact prints of human plasma are generated and checked with a red fluorescent Alexa-546 antibody, where several 30 μm lanes are seen **(A,B)**. These microcontact prints are then connected to (half)brain slices at the hippocampal level and cultured for 2–4 weeks **(C,D)**. Iba1-positive microglia migrate out from the brain slices along the lanes and can be quantified **(E,F)**. Plasma from healthy controls and plasma from Alzheimer’s patients have different effects on the migration pattern of round-amoeboid microglia, and their protein pattern can be differentiated with differential mass spectrometry. This approach may allow the identification of new putative biomarkers. Scale bar = 260 μm **(A)**; 870 μm **(D)**; 150 μm **(F)**.

To decrease the amount of collagen in brain slices, we developed a microcontact printing (μCP) method (Steiner and Humpel, 2023). Using a master plate with respective stamps, small lines (30 μm width) can be printed directly onto the semipermeable membrane with any factor of interest (Figure 5). In our study, we loaded human plasma onto these lanes, coupled them to mouse brain slices, and visualized the migration of cells outside the slices (Steiner and Humpel, 2023). We hypothesized that the plasma of patients with AD contains factors that inhibit or activate mouse-derived cells compared to plasma from healthy controls.

Microcontact printing allows the transfer of proteins or peptides onto membranes in a sub-μm resolution. For printing, polydimethylsiloxane (PDMS) stamps were fabricated from a master mold with the desired lane pattern. For exploration of novel biomarkers, 30 μm lanes were printed onto cell culture membranes loaded with collagen. Collagen hydrogel-based microcontact prints allowed proteins to be gradually released into organotypic brain slices. Collagen also supports cell migration, attachment, differentiation, proliferation, and survival. To search for plasma biomarkers, plasma from patients with AD can be μCP together with collagen onto membranes as 30 μm lanes. Briefly, 100 μL of plasma is lyophilized and resuspended in the collagen-PEG solution and then printed as 30 μm lanes onto the membrane and ultraviolet (UV) sterilized. We believe that a short exposure (10 min) to UV light does not affect the proteins loaded in collagen, although we do not have data or evidence to support this claim, and further studies are needed to prove it. The next day, brain slices were placed onto these plasma prints in the culture inserts. After 2–4 weeks of culture, the slice-derived cells that migrated along the lanes or formed vessels along the lanes were quantified.

Using this method with subsequent differential mass spectrometry, we identified new putative biomarkers in AD plasma (Yilmaz et al., 2024; Steiner et al., 2024). First, we showed that the plasma of patients with AD inhibits the migration of round-amoeboid Iba1 positive microglia (Steiner et al., 2024). This process could be mediated via mannose-binding protein C, macrophage receptor MARCO, complement factor H-related protein-3, and C-reactive protein. Second, we showed that plasma of patients with AD enhances the formation of myelin oligodendrocyte glycoprotein+ dots along nerve fibers, possibly involving aldehyde-dehydrogenase 1A1, alpha-synuclein, and protein S100-A4 (Yilmaz et al., 2024). Finally, we showed that plasma of patients with AD increases migration of laminin+/lectin+ endothelial cells, possibly via modulation of C-reactive protein, basigin, and trem-like transcript 1 protein (Yilmaz et al., 2025).

### Experimental applications using organotypic brain slices

In this review, I discuss how distinct pathologies of AD can be studied in organotypic brain slices: manipulation of β-amyloid plaques, phosphorylation of tau, inflammation and reactive gliosis, cell death and protection of cholinergic neurons, and immune responses, as well as to find novel biomarkers. Table 1 provides a summary of how *ex vivo* brain slices can help understand AD pathologies.

**Table 1:** Comparison between human Alzheimer’s disease (AD) and organotypic mouse brain slices.

	Human Alzheimer’s disease	Organotypic mouse brain slice
Age	Sporadic AD starts in humans at age >65 years, while familial AD is observed in humans at age >20 years.	Organotypic brain slices, prepared from postnatal day 8–10 mice, can be cultured for several weeks, but they do not mimic the adult age. While slices from adult mice can be prepared, the brain cells do not survive well.
Cognition	Memory is markedly impaired in patients with AD.	Memory and cognition cannot be studied in organotypic mouse brain slices.
Genetic AD	Several gene mutations are observed in familial AD, such as amyloid precursor protein (APP) or presenilins.	Organotypic mouse brain slices can be cultured from adult transgenic mice, such as mice with a point mutation in the Swedish-Dutch-Iowa APP gene (APP-SweDI), for example.
Sporadic AD	Sporadic AD progresses over 10–30 years; however, the reasons for AD remain unknown. Several risk factors may play a role, such as high blood pressure or high cholesterol, or genetic risk factors, such as apolipoprotein E4 or TREM.	Many different risk factors can be added to the medium of organotypic brain slices and may mimic the sporadic AD form; however, to date, no model has been established. Such risk factors include metals, different intracellular pathway stimuli, or any drug or protein that affects cellular metabolism.
β-amyloid plaques	In severely affected AD brains, diffuse and neuritic plaques are made of toxic β-amyloid (1–42) peptide.	Neuritic plaques can be studied in transgenic mice, as well as their degradation using ß-amyloid degrading enzymes, such as neprilysin, for example. To date, determining the reason why endogenous neuritic plaques are generated in mouse slices has not been fully elucidated.
Tau pathology	The tau protein plays a role in intraneuronal transport and is markedly hyperphosphorylated in AD at several sites (up to 40). This pathology causes the formation of intraneuronal fibrillary tangles.	In mouse brain slices, hyperphosphorylation of tau can be studied using drugs such as okadaic acid. Protein kinases (e.g., GSK-3ß) that phosphorylate tau or phosphatases that dephosphorylate tau can be studied. To date, the generation of endogenous neurofibrillary tangles in mouse brain slices has not been fully clarified or observed.
Neurodegeneration	Acetylcholine is the major neurotransmitter involved in memory, and cholinergic neurons degenerate in AD. These neurons are located in the septum or nucleus basalis of Meynert and project into the hippocampus or cortex, respectively.	Cholinergic neurons degenerate in brain slices and are protected by adding nerve growth factor (NGF) to the medium. The mechanism and protection of these neurons can be investigated in mouse brain slices. Co-cultures are prepared from two functionally connected brain areas. Other different protective molecules or their receptors, such as p75^NTR^ or tropomyosin receptor kinase A, can also be studied.
Inflammation	Severe inflammation occurs in AD, with enhanced expression of interleukin-1ß, transforming growth factor, or tumor necrosis factor-α, for example.	Many different inflammatory stimuli can be added to the culture medium of mouse brain slices to mimic the inflammatory situation in AD.
Blood–brain barrier (BBB)	In AD, the BBB is permeable, and blood-derived toxic molecules can enter the brain.	An artificial BBB cannot be incorporated into mouse brain slices, nor can it mimic a permeable BBB or the diffusion/transport of substances via the BBB into mouse brain slices.
Vessel pathology	In the AD brain, vessels are damaged and dysfunctional, and angiogenesis and reformation of vessels are observed.	The vessels are well preserved, but they are not functional. These vessels are more appropriately named ‘vessel-like.’ However, vessels can re-grow or grow together when stimulated with angiogenic factors, such as fibroblast growth factor-2. Laminin is a well-known marker to label vessels.
Cerebral amyloid angiopathy (CAA)	In the AD brain, severe CAA is observed, where the small peptide ß-amyloid (40), i.e., with 40 amino acids, is deposited in vessels and capillaries. The clumping of vessels may cause ischemic silent strokes and loss of energy supply to the brain.	A CAA study is possible only if ß-amyloid (1–40) is infused into anesthetized adult mice via the heart. A mouse brain slice with ß-amyloid CAA is then compared to AD CAA.
Reactive astrogliosis	Reactive astrogliosis is a pathology observed in the AD brain and is visualized with enhanced expression of glial fibrillary acidic protein (GFAP). These GFAP+ astrocytes are mainly observed around the neuritic ß-amyloid plaques in the AD brain.	Astrocytes are markedly expressed in organotypic mouse brain slices. Reactive astrogliosis can be stimulated using different toxins or oxygen–glucose deprivation (OGD). Reactive astrogliosis can also be observed in slices around ß-amyloid plaques cultured from transgenic mice.
Microglia dysfunction	Microglia are the immune cells of the brain and are functionally active in eliminating debris and ß-amyloid plaques. Microglia, round-amoeboid in shape, migrate to plaques or differentiate into macrophages to eliminate neuritic plaques. Microglia activity is markedly reduced in severe AD.	Microglia survive in brain slices and can be studied using Iba1 or CD11b antibodies. The addition of lipopolysaccharide (LPS) stimulates microglia to migrate, and the addition of granulocyte macrophage colony-stimulating factor activates differentiation. Microglia can be damaged to mimic impaired function in AD.

AD, Alzheimer’s disease; APP, amyloid-precursor protein; BBB, blood–brain barrier; CAA, cerebral amyloid angiopathy; CD11b, cluster of differentiation microglia antibody; GFAP, glial fibrillary acidic protein; GSK-3ß, glycogen synthase kinase-3ß; Iba1, microglia-specific antibody; LPS, lipopolysaccharide; NGF, nerve growth factor; OGD, oxygen–glucose deprivation; SweDI, Swedish-Dutch-Iowa mutation.

### Limitations of brain slices

Organotypic brain slices have several limitations. It is a disadvantage that the complex brain pathology of AD cannot be studied in slices at the same time. To date, we have not been able to stimulate slices to produce β-amyloid plaques or to test the hyperphosphorylation of tau and its complex sporadic cascade of events. We do not yet have a brain slice culture system for modeling all pathologies of AD and their time-dependent progression. We also lack a functional BBB connected to brain slices to study vascular-related pathologies and influx and efflux processes in the blood and cerebrospinal fluid. Furthermore, postnatal slices do not mimic complex age-related diseases, as sporadic AD is seen in humans aged > 65 years, although the pathology begins 30 years prior. While there are limitations regarding AD pathology, the slice technology also has technical limitations. It is very difficult to establish and culture brain slices and requires at least 2–3 months of experience to learn and handle this technique. The main challenge is producing slices that flatten during culture and display high survival rates in all brain cells. The addition of serum and growth factors may selectively enhance the survival of specific cells. However, the major disadvantage is that we and others are unable to culture adult slices with healthy neurons or other brain cells. Further study is necessary to optimize the long-term survival (>2 weeks) of brain cells in adult slices, especially when considering adult human tissues.

### Outlook and translation to humans

In summary, organotypic brain slices offer an excellent ex vivo model for mimicking different aspects of neurodegenerative diseases, including AD, Parkinson’s disease, and other brain disorders. Much research has been conducted to improve this model.

1. The culturing of brain slices on Millipore inserts is expensive, and thus far, Merck inserts are the best choice. We have already reduced costs by reusing undamaged inserts and using an extra membrane on top of the inserts. Pre-cut extra membranes are also cost-effective. Alternatively, we cut the membrane inserts from a roll, which markedly reduces cost. To date, we have developed novel “ring-inserts,” where we glued a membrane on plastic rings and culture slices. This is the cheapest and most reproducible method, and we have demonstrated the survival of cholinergic and dopaminergic neurons (Gern et al., submitted 2025).
2. These “ring inserts” were tested for live-cell imaging of brain slices. A disadvantage of slices is that they grow on membrane inserts and cannot be investigated without cutting the membranes. However, the use of additional membranes can overcome this problem. Using our novel “ring-inserts,” we can investigate organotypic brain slices directly under the microscope for hours or weeks. A recent report from our lab showed that astrocytes can be fluorescently labelled with GFAP and vessels with laminin using microcontact printing, and can be investigated over weeks using live cell imaging techniques (Humpel, 2025).
3. The major challenge is culturing brain slices from adult mice or rats. This will revolutionize experimental culture models, as such models are more likely to mimic the adult situation of AD or other brain disorders. To date, the survival of adult slice cells has been very low, and the conditions under which adult slices show high viability must be developed. Interestingly, slices from brains taken from mice older than 12–14 days do not flatten well, and brain cells do not survive well in such slices. It is unclear why such slices show a lower viability at this age, and we recommend using brains from postnatal day 8–10 for optimal culturing.
4. Finally, it would be extremely interesting to prepare and culture brain tissue from human brains and translate the findings to a real human AD situation. Definitely, the pioneering study of Mendes et al. (2018) may offer such a novel way to culture human brain slices at least for a short period of time. Brain slices should offer a way to culture brain sections after biopsy or from postmortem brains and culture slices over weeks, and to investigate their pathologies. Modern computer-assisted techniques and artificial intelligence may help optimize this technique in the future.
